# Physical Activity as a Modern Intervention in the Fight against Obesity-Related Inflammation in Type 2 Diabetes Mellitus and Gestational Diabetes

**DOI:** 10.3390/antiox12081488

**Published:** 2023-07-25

**Authors:** Katarzyna Piotrowska, Katarzyna Zgutka, Marta Tkacz, Maciej Tarnowski

**Affiliations:** 1 Department of Physiology, Pomeranian Medical University in Szczecin, al. Powstancow Wlkp. 72, 70-111 Szczecin, Poland; 2 Department of Physiology in Health Sciences, Faculty of Health Sciences, Pomeranian Medical University in Szczecin, Zolnierska 54, 70-210 Szczecin, Poland

**Keywords:** gestational diabetes mellitus, type 2 diabetes mellitus, physical activity, inflammation, oxidative stress

## Abstract

Diabetes is one of the greatest healthcare problems; it requires an appropriate approach to the patient, especially when it concerns pregnant women. Gestational diabetes mellitus (GDM) is a common metabolic condition in pregnancy that shares many features with type 2 diabetes mellitus (T2DM). T2DM and GDM induce oxidative stress, which activates cellular stress signalling. In addition, the risk of diabetes during pregnancy can lead to various complications for the mother and foetus. It has been shown that physical activity is an important tool to not only treat the negative effects of diabetes but also to prevent its progression or even reverse the changes already made by limiting the inflammatory process. Physical activity has a huge impact on the immune status of an individual. Various studies have shown that regular training sessions cause changes in circulating immune cell levels, cytokine activation, production and secretion and changes in microRNA, all of which have a positive effect on the well-being of the diabetic patient, mother and foetus.

## 1. Introduction

Diabetes is a central healthcare concern, with 537 million people affected worldwide in 2021 according to data from the International Diabetes Federation [1]. By 2040, it is predicted that more than 700 million people will develop this condition and 20 million people will die from diabetes [1]. Diabetes is characterised by elevated blood glucose levels (hyperglycaemia) due to insufficient insulin production or the presence of insulin resistance (IR). The glycaemic criteria of diabetes according to the American Diabetes Association (ADA) are a fasting plasma glucose level of at least 126 mg/dL (7.0 mmol/L) or at least 200 mg/dL (11.1 mmol/L) 2 h after the standard oral glucose tolerance test (75 g anhydrous glucose) [2].

Diabetes is a heterogeneous metabolic disease with multiple causes [3] and is accompanied by severe complications, such as heart disease, stroke and renal failure. Type 1 diabetes is caused by autoimmune-mediated pancreatic cell destruction, usually diagnosed in childhood by the presence of islet cell antibodies, which are absent in type 2 diabetes mellitus [4]; it accounts for 5–10% of all cases of diabetes mellitus. T2DM is the most common form of diabetes and is influenced by obesity, age, and environmental and genetic factors [5]. Poor diet, insufficient physical activity and, the consumption of alcohol, tobacco and other drugs, in addition to a lack of sleep and rest, all affect the metabolic processes throughout the body and are important predictors of obesity and T2DM [6,7]. The global incidence of overweight and obesity is continuously rising and almost one-third of the current world population is now considered overweight or obese [8].

Gestational diabetes mellitus (GDM) is a common metabolic condition that is present in pregnancy and shares many features with T2DM, including glucose intolerance and insulin resistance [9,10]. It is defined as any degree of glucose intolerance of variable severity with onset or first detected during pregnancy and usually resolves not long after delivery [11]. World Health Organization (WHO) guidelines for diagnosis of GDM indicate the presence of GDM with >126 mg/dL blood glucose or >140 mg/dL blood glucose after 75 g glucose challenge [11]. Generally, the frequency of GDM may range from 5 to 20% of all pregnancies, depending on the population studied and the diagnostic tests employed [12,13]. The maternal hormonal and metabolic alterations associated with GDM profoundly modify the in utero environment, leading to an abnormal pattern of foetal growth [14], with an increased risk of developing glucose intolerance and obesity in adolescence [15,16]. Women with GDM have an increased risk of prenatal morbidity and significantly elevated (approximately seven-fold increase compared to non-GDM pregnancies) risk of T2DM in later life [17].

In physiology, pregnancy-induced insulin resistance has great importance as it ensures a proper glucose supply for foetal growth and development by decreased blood glucose transportation to muscles [18]. As the pregnancy progresses, insulin secretion has to be increased 2–4 times to maintain glucose levels within normal limits. In GDM, pregnant women are unable to augment insulin production to compensate for their increased resistance to insulin [19]. Defects in insulin sensitivity are, together with a larger pre-pregnancy body weight, the hallmarks of GDM [20]. Obese women (BMI > 30) have a three-fold increased risk of developing GDM compared to non-obese women and overweight women (BMI ≥ 25) are 1.8–6.5 times more vulnerable to GDM [21]. Additionally, it has been shown that an interpregnancy BMI gain is associated with a higher risk of GDM during subsequent pregnancies [22,23,24]. The Hyperglycaemia and Adverse Pregnancy Outcome (HAPO) study shows a direct relationship between BMI and pregnancy complications (preeclampsia, caesarean section, macrosomia—higher neonatal birth weight, premature birth and stillbirth) [25].

## 2. Hyperglycaemia and Inflammation—Molecular Aspects of Inflammation in Diabetes

When energy consumption is not adequate for energy expenditure and when the pancreatic response is not sufficient to overcome rising carbohydrate levels and progressing insulin insensitivity, this results in a state of hyperglycaemia. This chronic energy imbalance culminates in obesity and overweight and the accumulation of the excess of fat in adipose tissue; the metabolism of carbohydrates, fat and protein is disturbed and the condition worsens [26]. Hyperglycaemia is known to cause immune dysfunction, adversely affecting leukocyte function and leading to the increased production and secretion of cytokines, such as IL-6, IL-1β and TNF-α, leaving diabetic patients more susceptible to infections and related comorbidities [27]. This state of chronic, low-grade inflammation is a crucial factor contributing to the development and progression of diabetes. Severe obesity (BMI > 30) is associated with elevated C-reactive protein (CRP), an objective marker of inflammation [28]. A high or very high BMI is correlated with an increased inflammatory status, not only in diabetic patients but also in normoglycaemic or pre-diabetic hyperglycaemic individuals [29]. Many reports show elevated levels of proinflammatory cytokines in T2DM. Pancreatic islet inflammation leads to β-cell dysfunction and the local influx of macrophages [30]. The pharmacological approach focuses on the use of anti-inflammatory drugs to support T2DM treatment (thiazolidinediones, sulphonylureas, aspirin, statins—reviewed in [31]).

During pregnancy, a state already affected by immunological alterations, a further imbalance in innate and adaptive cellular responses will pose additional health risks in women with a diagnosis of GDM. Despite ongoing progress in our knowledge on extra and intracellular pathways, the pathophysiology of GDM is not fully understood and role of immune dysregulation as a causative mediator in GDM pathophysiology is currently unclear. Multiple hypotheses link aberrant hormone production in the placenta to maternal metabolic dysfunction and diminished insulin functionality [20,32] or GDM development due to the aberrant adaption of the maternal immune system to pregnancy and the increased release of circulating inflammatory factors [33,34]. The evoked immune pathway dysregulation and its consequences (oxidative stress and subsequent endothelial dysfunction, vasculopathy, together with immune cell infiltration of visceral adipose tissue) produce a state resembling that seen in T2DM patients, characterised by the pathological disruption of insulin signalling and insulin resistance [35].

Since the mid-20th century, we have been aware that diabetes, obesity and inflammation are linked. It was shown that treatment with high doses of nonsteroidal anti-inflammatory drugs decreased glucosuria in patients presumed to have T2DM (reviewed in [36,37]). The excess of nutrient flux, hyperglycaemia and elevated free fatty acids stimulates glucose utilisation along with alterations in oxidative phosphorylation and the induction of oxidative stress [27,38,39,40,41]. It is not known whether inflammation induces hyperglycaemia and IR or if the reverse is true-hyperglycaemia triggers inflammation. It has been concluded, however, that obesity and the accompanying low-grade inflammation are the major mechanisms of diabetes and insulin resistance induction [5]. The formation of this proinflammatory environment, specifically in adipose tissue and the pancreas, as well as in the skeletal muscle, liver, gastrointestinal tract or placenta (in the context of GDM), attract and stimulate a number of inflammatory cells, such as neutrophils, basophils, M1 and M2 macrophages, TH1, TH2, Treg cells, CD8+ cells and B cells; in turn, these release chemokines, cytokines and adipokines, which modulate inflammatory responses [27,42,43,44,45]. The pioneering studies by Hotamisligil and colleagues suggested that immunomodulatory treatments may improve glycaemia, β-cell function and/or IR in patients with T2DM [46,47]. It was this important observation that proinflammatory TNF-α was highly expressed within adipose tissue in several rodent models of obesity and, when TNF-α was neutralised, insulin action was enhanced [46,47]. Subsequent studies by these researchers demonstrated that genetically obese ob/ob mice with targeted mutations in TNF receptors display an improved insulin sensitivity relative to ob/ob control mice [48]. Similarly, the neutralisation of TNF-α with antibodies improved glycaemia in obese rodents, making it a potential therapeutic target in T2DM treatment [46]. Furthermore, TNF-α mRNA expression was shown to be increased in the adipose tissue of obese hyperinsulinaemic human subjects [49]. Recent meta-analysis including more than 20 studies has shown a significant increase in blood serum TNF-α levels in people with diabetes [49]. Despite overwhelming evidence in favour of TNF-α having a critical role in regulating inflammation and insulin-action, the translation of basic research findings with TNF-α-targeted neutralisation in diabetes showed disappointing results. However, recent studies have shown that the development and application of new neutralising antibodies to both TNF-α and its receptors may improve glucose metabolism and insulin resistance in patients with other inflammatory conditions [50,51,52,53,54].

The above-mentioned studies and those showing the involvement of other pro-inflammatory molecules like IL-1β, IL-6, IL-13, IL-33 and their receptors [55,56] constitute a proof of concept that chronic inflammation is implicated in the pathophysiology of diabetes; therefore, targeting inflammation may ameliorate diabetes and insulin resistance, preventing its progression and any vascular complications. The presence of subclinical and chronic inflammation, accompanied by obesity and IR in diabetes, means that this pathology is considered an inflammatory chronic disease. This state of low-grade inflammation was recently called “metaflammation” [57,58]. The studies, together with the subsequent identification of two key molecules downstream of the transmembrane cytokine receptors, namely the inhibitor of kappa B kinase (IKKb), an important element of NF-κB pathway, and c-Jun NH2-terminal kinase (JNK), were critical in our understanding of the link between the processes involved in nutrient overload, obesity and impaired insulin action with immune-related intracellular signal transduction pathways [45,55,59,60].

This picture has, however, many different aspects and is overly complicated, producing multiple autocrine and paracrine loops of ongoing stimulation. Nonetheless, we can name several factors involved in the mechanisms of this unresolved chronic inflammation.

IL-1β is one of the most central players in the pathogenesis of T2DM. Even low concentrations of IL-1β are selectively toxic for insulin-producing pancreatic β-cells [38,61]. Binding to the IL-1 receptor 1 (IL-1R1) activates NF-κβ pathways which leads to the increased production and release of other inflammatory mediators, such as TNF-α, IL-6 and IL-1β itself, thus initiating a self-amplifying, autocrine cytokine network. The control of IL-1β generation is tightly regulated and proceeds in two steps including the initial stimulation of IL-1β expression by a pro-inflammatory signal with subsequent storage of inactive pro-IL-1β in the cell. The second step involves the production of active, mature IL-1β by cleavage of its inactive precursor by caspase-1, which is activated in a large cytoplasmic multiprotein complex called the inflammasome [62].

Inflammasomes are important components of the innate immune response. Microbial products (pathogen-associated molecular patterns (PAMPs)) or endogenous molecules (danger-associated molecular pattern (DAMPs)) are recognised by innate pattern recognition receptors (PRRs). Endogenous, non-microbial DAMPs are considered metabolic danger signals, such as urate, cholesterol crystals, extracellular ATP, certain fatty acids and islet amyloid peptides that may accumulate in obesity and hyperglycaemia. Various types of PRRs have been identified so far, like retinoic acid-inducible gene I-like helicases (RLHs), toll-like receptors (TLRs) and nucleotide-binding oligomerisation domain-like receptors (NLRs), including the well-studied NLR family, pyrin domain-containing 3 (NLRP3) cytosolic protein [63,64,65]. Activated NLRP3 interacts with ASC (the adapter protein apoptosis-associated speck-like protein). Subsequently, the caspase recruitment domain (CARD) of ASC binds to the CARD domain on procaspase-1, forming the NLRP3 inflammasome [62,66,67]. This leads to the generation of active caspase-1, which induces the secretion of IL-1β and IL-18 [62,66,67]. The role of NLRP3 inflammasome in the pathogenesis of obesity was supported by data showing that the genetic deletion of Nlrp3 and Asc in high-fat diet fed mice results in improved glucose tolerance and IR [39,44,68,69]. In addition, the gene knockout animals have decreased circulating IL-18 and reduced adipose tissue IL-1β, which are markers of caspase-1 activation [44]. The NLRP3 inflammasome activation can be prevented by reducing caloric intake and represents another mechanistic link between obesity, IR and T2DM [70].

Hyperglycaemia together with glucotoxicity and lipotoxicity triggers a number of pathological processes like oxidative and endoplasmic-reticulum (ER) stress, raised lipid levels and amyloid deposition; these are involved in cellular dysfunction and trigger an inflammatory response [7,71]. Among several sites of inflammation in metabolic diseases, adipose tissue is a large contributor to circulating proinflammatory cytokines during obesity [72]. Adipose tissue hormones are known as adipokines; these molecules are secreted into the circulation and regulate glucose and lipid metabolism and insulin sensitivity [72,73]. In obesity and metabolic syndrome, a highly inflammatory status is induced by the infiltration of inflammatory cells into the adipose tissue, especially activated macrophages which are functionally and numerically dominant [74]. In obese mice, the number of macrophages in adipose tissues is estimated to be increased by around five times [75]. Also, adipose tissue macrophages of obese mice have a pro-inflammatory, classical (M1) phenotype, highly expressing the NLRP3 inflammasome [76]. These classically activated macrophages secrete pro-inflammatory cytokines, which induce insulin resistance via IKKβ and JNK by the serine phosphorylation of IRS proteins [74]. Under these conditions, the adipose tissue produces proinflammatory adipokines (TNF-α, IL-6, MCP-1, lipocalin-2 and resistin) [77,78] and the production of immune-modulatory adiponectin is markedly reduced. Another aspect of adipose tissue inflammation in obesity is local hypoxia caused by the rapid expansion of adipose tissue with insufficient vascular adaptation [79].

Similar to that seen in T2DM, maternal adipose tissue dysfunction seems to be implicated in the pathophysiology of GDM. A strong association has been found between maternal visceral adipose tissue mass and GDM diagnosis [80,81]. Insulin-mediated suppression of lipolysis, elevated FFA levels and glucose production and severe insulin resistance are common in GDM [82]. Rising inflammatory status together with dysregulation of the cytokine network has a significant negative effect on maternal insulin function and glucose levels, causing inflammation in the placenta [83]. The decreased secretion of anti-inflammatory adiponectin and the increased secretion of pro-inflammatory leptin and IL-6 is reported in GDM, similar to high concentrations of TNF-α and IL-1β due to hyperglycaemia [84,85,86,87]. Due to hyperglycaemia, the increased infiltration and activation of neutrophils, macrophages, and B and T lymphocytes is observed [32]. At the same time, the transport of nutrients in the placenta is affected by changes in the expression of transporters. The increased transport and hyperglycaemic conditions in the GDM placenta stimulate a pro-inflammatory response in human trophoblasts [84,86]. In GDM term placentas, an increased number of macrophages, and levels of IL-1β, IL-6, MCP-1, leptin, TNF-α, IL-7, IL-8 and TRL-4 gene expression is noted [43,83,88]. This may result in villous immaturity, villous fibrinoid necrosis, chorangiosis and increased angiogenesis, with an increase in overall size [89]. If not controlled, the overall augmented cytokine production (stimulation of NLRP3 inflammasome and generation of IL-1β and IL-18 inflammatory cytokines [90]) during GDM may not only affect the mother but also compromise the normal development of the growing foetus, with an increased risk of serious complications for the neonate [32].

## 3. Fight against Chronic Inflammation

It is known that a sedentary, non-active lifestyle is associated with an increased cardiometabolic risk, obesity and T2DM, as well as with chronic obstructive pulmonary disease, colon and breast cancer, dementia and depression. Lifestyle changes such as increasing physical activity (PA) and decreasing caloric excess are essential for the proper control of body weight and hyperglycaemia [91,92]. A gradual loss of weight of up to 16% of the original body weight is sufficient to improve β-cell function and insulin sensitivity the in adipose tissue, liver and skeletal muscle [93].

**Figure 1:**
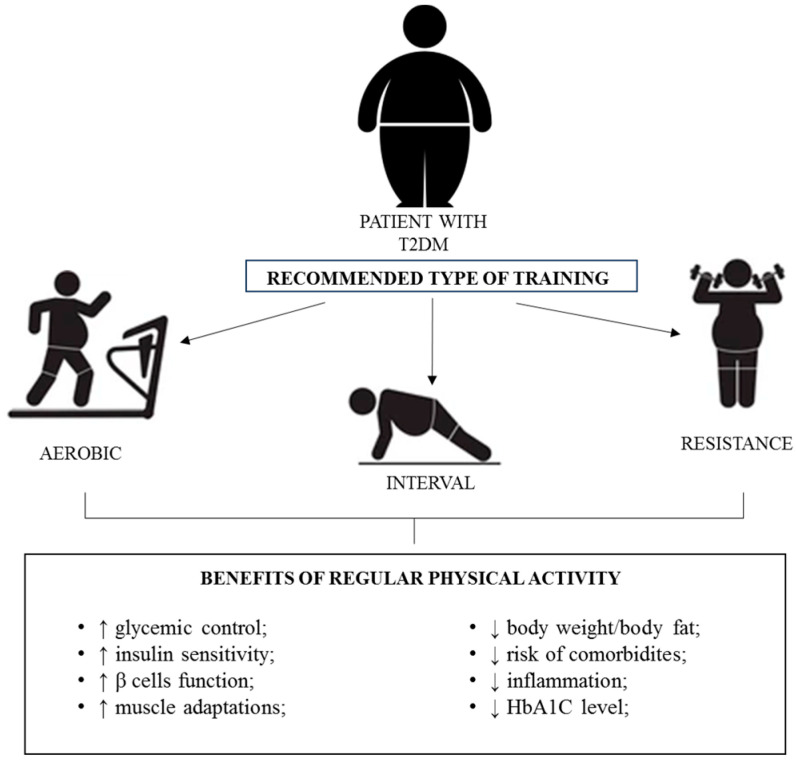
Effects of PA in T2DM patients. Arrows up—improvement; arrows down—decrease.

PA is crucial for the maintenance of health. The World Health Organisation (WHO) recommends a combination of moderate to vigorous-intensity PA for substantial health benefits. Despite outdated worries related to exercise-induced injury or negative foetal and maternal effects, pregnant women are encouraged to include aerobic exercise in their regular, daily activities [94,95]. The American College of Obstetricians and Gynaecologists and the American Diabetes Association (ADA) advise GDM patients to implement 30 min or more of moderate exercise each day [96,97,98]. Regular moderate-intensity PA is considered a non-pharmacological “adjunctive long-lasting anti-inflammatory therapy”. This intervention strategy has high anti-inflammatory, antioxidant and immunosurveillance potential that modulates carbohydrate metabolism, atherosclerosis and other disease processes [99,100,101,102]. Importantly, PA may play a crucial role in treating and improving the quality of life of T2DM patients via several effects (Figure 1).

In view of the huge negative effects of diabetes on individuals and society, it is important to update and explore new potential treatments for the disease. In this review, we would like to provide an explanation and rationale for the importance of physical activity in the fight against the huge social, economic and health burden caused by diabetes, emphasising the anti-inflammatory effect of physical exercise at the molecular level in the context of T2DM and GDM.

## 4. PA and Inflammation in Obesity, Metabolic Syndrome and T2DM

Physical training or general PA has an influence on the immunological status of the individual. Regular training sessions and single bouts of exercise cause changes in circulating immunological cell levels, cytokine production and secretion and microRNA release [103].

The chronic exposure of beta-cells to circulating inflammatory factors such as IL-1β, IFN-γ and TNF-α cause their dysfunction and inhibit insulin secretion [104]. It is known that islet inflammation is a key factor in T2DM pathogenesis [30]. Immune cell infiltration, amyloid deposition (IAPP), cell death, fibrosis, greater oxidative stress and increased inflammation markers are the most common pathological changes in the pancreatic islets of T2DM patients. It was shown in rodent models as well as in patients with T2DM that the number of intra-islet macrophages is increased [30] and the hyperglycaemic conditions promote the differentiation of these cells into pro-inflammatory phenotypes [105].

Using the ZDF rat as an animal model of obese T2DM, de Lemos et al. [106] has shown that regular exercise training was able to prevent the accumulation of pro-inflammatory cytokines (IL-6 and TNF-α) in the pancreas and improves the whole-body insulin sensitivity. Moreover, lifelong physical training improved the body adiposity, plasmatic insulin concentration and macrophage immunostaining in the pancreas [106]. Regular physical training also promoted the increased expression of haem oxygenase-1 (HO-1) in the pancreas. HO-1 is a crucial cytoprotective enzyme which plays pivotal role in regulating redox homeostasis and has strong antioxidative and anti-apoptotic properties [106]. Comparable results were obtained by Carvalho et al. [107], who demonstrated that long-term (60 weeks) exercise in male Sprague-Dawley rats caused a significant reduction of IL-1β in the circulation and IL-1β, TNF-α, TGF-β and the NF-κB p65 subunit in the pancreas. In addition, macrophage infiltration in the islets was also attenuated by exercise [107]. As mentioned above, β-cell apoptosis is also observed in human pancreatic sections and post-mortem islet grafts in hyperglycaemic conditions [108]. Exercise positively influences β-cells, preventing apoptosis or increasing proliferation in islets, which leads to better control of the number of β-cells in the diabetic pancreas and increases the number of small islets [109,110].

As noted above, the NLRP3 inflammasome is strongly associated with sterile inflammation [111]. Researchers are investigating its role in diabetes and in diabetic complications [112,113,114]. Lee et al. demonstrated increased mRNA and protein expression of NLRP3, apoptosis-associated speck-like protein containing a CARD (ASC) and proinflammatory cytokines in monocyte-derived macrophages (MDMs) from patients with newly diagnosed T2DM compared with healthy controls [112]. Zhang et al. showed that insulin resistance, liver injury and NLRP3 inflammasome activity were higher in 50 pre-diabetic patients than in the normal control group [111].

The expression of NLRP3 can be modulated by exercise interventions and depends on the exercise regimen and intensity [114]. The NLRP3 inflammasome seems to be activated during the early stage of acute exercise, as well as during the recovery period [114]. Khakroo Abkenar et al. [115] showed that chronic exercise with moderate intensity significantly reduced the expression of the NLRP3 gene and serum levels of IL-1β and IL-18 in a group of healthy, young men. In the case of chronic exercise with high intensity, a significant increase in these parameters was observed [115]. In another study, 8-week resistance training prevented NLRP3 inflammasome activation by decreasing its expression in peripheral blood mononuclear cells (PBMCs) obtained from old, healthy women and men [116].

It is known that IL-6 is secreted from contracting skeletal muscle during and after physical activity in humans [117,118]. This important myokine increases the circulating levels of incretin glucagon-like peptide-1 (GLP-1), which improves glucose-stimulated insulin secretion, insulin gene expression and biosynthesis [118]. In addition, GLP-1 protects β-cells from apoptosis and promotes β-cell growth [117]. Moreover, IL-6 induces an increase in the production of IL-1RA and IL-10, thus exerting a systemic anti-inflammatory effect [118].

In groups of obese postmenopausal women, PA helped to decrease subclinical inflammation, improved fasting glucose levels and increased insulin sensitivity [119,120]. Similarly, in a study by Bruun and co-workers [121], moderate PA improved body weight, and lowered waist circumference (WC) and total body fat mass. Examinations of the adipose tissue (AT) and skeletal muscles (SM) showed decreased macrophage infiltration in AT and decreased mRNA levels of IL-6 (AT, MS) and TNF-α (AT), which leads to the conclusion that a decrease in AT content causes a reduction in inflammation [121].

It is important to note that physical activity increases glucose uptake and utilisation by the skeletal muscles. This is possible due to the insulin-independent activation of glucose transporter 4 (GLUT4) expression and translocation to the muscle membrane after exercise. The increased transport of carbohydrates to the skeletal muscles lowers blood glucose levels [122]. Apart from increasing muscle glucose uptake, exercise also increases muscle insulin sensitivity in the post-exercise period [123]. With regard to the interstitial glucose concentration, microvascular perfusion is particularly relevant as correlative evidence supports a connection between insulin sensitivity and microvascular perfusion [123]. Various types of PA not only increase glucose transport and utilisation in skeletal muscles, but also induce many modifications and adaptations in the skeletal muscles itself. It is already established that PA increases muscle strength, endurance and the quality of fibres and improves neuromuscular functions [124,125]. In single-bout acute cardiopulmonary exercise, Contrepois and co-workers [126] showed step-by-step changes in inflammatory status in insulin-resistant, obese subjects. After exercise, PA inflammatory markers and cells increased rapidly (2–15 min after the PA), but most of them return to their basic values within 60 min of recovery and only Th1 and Th2 increased activation pathways persisted 1 h post-exercise [126]. In this interesting study, it was noted that the parameters of oxidative stress were affected by the accumulation of myeloperoxidase (MPO), which is a product of activated neutrophils upon skeletal muscle damage [126].

It has been found that, in addition to the type of exercise and its intensity, the time of day also has great importance. Savikj et al. [127] performed a comprehensive analysis of the multi-tissue metabolomic and skeletal muscle proteomic responses to a short-term training regimen at different times of the day in men with T2DM. They showed that two weeks of afternoon high-intensity interval training (HIIT) increased skeletal muscle lipids and mitochondrial content to a greater degree than morning training. In another paper by the same group, it was shown that afternoon HIIT was also more effective than morning HIIT at improving blood glucose levels in men with T2DM [128]. Strikingly, morning HIIT had an acute, deleterious effect, increasing blood glucose [128].

The metabolic effects of PA in T2DM are connected to proper glycaemic control with appropriate glycated haemoglobin (HbA1c) levels. HbA1c is correlated with blood glucose and is a good predictor of the lipid profile. Its elevation (above 7%) is associated with cardiovascular disease (CVD) and stroke and is associated with an approximately 30% increase in all-cause mortality [129]. An increase in HbA1c affects erythrocyte physiology; it decreases cellular flexibility and increases the aggregation tendency, leading to the elevation of blood viscosity and vascular resistance. Additionally, high levels of HbA1c lower the oxygen-carrying capacity and promote hypoxia [129]. Moreover, evidence of a relationship between HbA1c and inflammation was previously reported in several studies [130]. Physical activity, specifically high intensity resistance training, is found to decrease levels of HbA1c in hyperglycaemic patients [131]. Of interest, this effect is less pronounced in less intense training [131]. On the other hand, in the independent studies of Savikj, Elsisi and Winding [127,132,133], high intensity interval training showed better results in reducing HbA1c than other exercise programs.

Recently, it has become evident that the hallmarks of diabetes, including, insulin resistance, impaired glucose tolerance and a proinflammatory environment, lead to endothelial dysfunction and accelerate atherogenesis [134]. Perks et al. [135] performed a cross-sectional study of 736 participants with T2DM and noted that the presence of peripheral artery disease may have been associated with lower physical activity levels and physical function. Moreover, it was already shown that PA, in different forms, ameliorates stiffness and the thickness of arteries in T2DM subjects and improves the control of blood pressure [136,137]. Lately, Garneau L et al. [138] compared the anti-inflammatory potential of 12 weeks of HIIT and moderate-to-vigorous intensity continuous training (MICT). They assessed the impact of these types of training programs on plasma cytokine concentrations in patients with coronary artery disease (CAD) with or without T2DM. Eleven targeted cytokines (IL-1β, TNF-α, CRP, secreted protein acidic rich in cysteine—SPARC, fibroblast growth factor 21—FGF-21, IL-6, IL-8, IL-10, IL-13, IL-15 and IL-18) were assessed. The co-occurrence of CAD and T2DM was associated with increased plasma IL-8. Both types of physical interventions reduced plasma FGF-21, IL-6, IL-8, IL-10 and IL-18 irrespective of T2DM status. In addition, HIIT and MICT resulted in similar reductions in circulating cytokines known to be increased in the context of low-grade inflammation in CAD patients, an effect that is more pronounced in patients with T2DM for FGF-21 and IL-6 [138].

The results of the National Health and Nutrition Examination Survey 1999–2004 (USA) showed that significant obesity was related to high CRP markers of inflammation [139]. de Lemos et al. showed that regular training induces a reduction in CRP levels, T2DM, insulin resistance and cardiovascular/cardiometabolic diseases [104].

In a group of men with MetS, 12 weeks of endurance training produced a decrease in WC and BMI and a decrease in MCP-1 and IL-8 levels in peripheral blood plasma [140]. In MetS women, PA affected the body composition (decreased body fat and WC), improved the systolic blood pressure and decreased inflammatory marker levels [140]. The results indicated a significant correlation of CRP levels with WC and glycaemia and, importantly, the TNF-α concentration with and glucose and HDL levels [141]. Older, adult, obese subjects with MetS participated in the PREDIMED-Plus study in which changes in inflammatory score, driven mostly by CRP level, were inversely associated with moderate-to-vigorous PA [142]. In a recent study, evidence of the effects of different long-term training interventions (aerobic, resistance and combined) and spontaneous physical activity in modifying CRP, IL-6, IL-18, IL-20, TNF-α and adipokines in patients with overweight or obesity with or without cardiometabolic diseases were investigated. The results suggested that all interventions, except spontaneous physical activity, were effective in lessening the inflammatory status [143]. A meta-analysis of twenty-six randomised controlled trials (RCTs) involving 1239 patients with T2DM demonstrated that aerobic exercise training significantly reduced the circulating levels of CRP [144]. A decrease of CRP and TNF-α was noted in most studies and one study showed a lack of change in CRP [145,146] or TNF-α [147]. These findings suggest a key role for exercise against local inflammation.

The systemic changes induced by exercise cause the appearance of plethora of different metabolites, which clearly indicate adaptation to exercise, and signify long-term changes at the cellular and tissular levels [148]. Currently, there is growing interest in small metabolites—“exerkines”, that produce long-term effects in a hormone-like fashion. Exerkines are defined as signalling moieties released in response to acute and/or chronic exercise, which exert their effects through endocrine, paracrine and/or autocrine pathways [148,149]. Many sources (organs, tissues and cells) secreting these molecules have been discovered, such as skeletal muscle (myokines), the heart (cardiokines), white adipose tissue (adipokines), brown adipose tissue (baptokines), liver (hepatokines) and neurons (neurokines) [149]. Recent studies have shown that during PA, along with an increase in blood lactate levels (and other molecules mentioned previously), N-lactoyl-phenylalanine (Lac-Phe) levels also increase [150]. Lac-Phe is a pseudo-dipeptide formed from lactate and phenylalanine, which shows a particularly pronounced increase in the peripheral blood during and shortly after PA. In interesting study by Li et al. [150] it was noted showed that macrophages, epithelial cells and other types of CNDP2+ (carnosine dipeptidase 2) cells secrete Lac-Phe. What is intriguing in the context of diabetes and obesity, Lac-Phe produced during PA may contribute to weight loss by acting as a signalling molecule that regulates food intake [148]. Monitoring the levels of Lac-Phe during exercise can be used as a promising tool to assess the individual response to PA, especially in overweight and obese people.

In conclusion, training programmes are now “prescribed” as an additional form of therapy. The American Diabetes Association (ADA) recommends that all individuals with DM engage in moderate intensity physical activity (MPA) for at least 150 min per week or vigorous-intensity physical activity (VPA) for at least 90 min per week; physical activity should be distributed over at least 3 days in a week, with no more than 2 consecutive days without physical activity [2]. Moreover, as shown above, there are many different clinical trials showing the positive effects of PA on inflammatory status in patients with obesity, metabolic syndrome and T2DM. Table 1 summarises some of them in groups of T2DM subjects.

As can be seen, the results are difficult to compare and do not give a single specific conclusion as the clinical trials utilised different exercise protocols and different training regimes (duration, number of sessions per week, different session durations). Most of the published studies that were considered clinical trials included diabetic, obese, middle-aged individuals, both males and females, without insulin treatment and comorbidities precluding PA. In only a few studies, healthy subjects were included as control groups; in others, results from T2DM patients without exercise interventions were presented for comparison. Training sessions included aerobic exercise and/or resistance training in some cases combined with specific diet or supplementation. Lifestyle interventions were most effective for T2DM remission for younger and early diagnosed patients [157]. There were studies showing remission or partial remission of T2DM after the lifestyle (exercise and diet) intervention [157,158]. In these studies, long-term follow-up showed a partial improvement in body mass control and increased physical fitness. Remission was achieved even if no further interventions were provided in the follow-up period [157]. In all of the presented trials, the authors showed changes in inflammatory status; however, various inflammatory markers were assessed.

## 5. Exercise During Pregnancy and GDM

Healthy lifestyle during pregnancy may decrease the risk of GDM development. In the “Omega study”, pregnant women were questioned about leisure time PA, diet, smoking and stress levels. In the group of 3005 women, 140 developed GDM, but only 7% reported all four healthy behaviours (sufficient level of PA, good diet, no smoking and low stress) [159]. Of these, 66% were physically active during pregnancy. The data indicated that women with no healthy lifestyle components had a 4.4 times greater risk for GDM than women with healthy lifestyle components. Each additional component was associated with a 21% lower risk for GDM [159]. The main conclusion from the study is that healthy behaviours like PA, a good diet, non-smoking and low stress are associated with each other and their influence is additive in the risk of GDM development [159].

As mentioned before, the ACOG recommends the performance of moderate aerobic exercise for 30 min or more, on most weekdays, with a pause of no longer than 48 h for improved glycaemic control [160,161]. The American Department of Health and Human Services (DHHA) recommends at least 150 min of moderate-intensity aerobic activity per week, avoiding the supine position and high fall risk sports like horseback riding. According to a recent study, only 15% of pregnant women are physically active [161]. With regard to pregnant women in the USA, 60% are not engaging in leisure time PA and those who are exercising decrease the amount of PA progressively from the first to the last trimester [162,163,164,165]. During pregnancy, a number of women self-reported as non-active doubled in the MoBa study, from 2% to 4% of pregnant women who participated in the study [165].

As regular training is beneficial for glucose homeostasis and weight control, regular PA is recommended for pregnant women at risk of or with GDM. Both forms of PA, aerobic and resistance exercise (AE and RE), are favourable in GDM. The engagement of large muscle groups during walking, stationary cycling and water aerobics, for example, is favourable for glycaemic control (decrease fasting glucose and HbA1c), while RE with i.e., elastic bands help with muscle mass improvement and reduces the need for insulin in GDM treatment [160].

There are several survey studies and clinical trials showing the impact of PA on glycaemic control, gestational weight gain and pregnancy outcomes in GDM. The results are often difficult to compare as studies have been performed with different groups of participants (without GDM, with a risk of GDM, with developed GDM) and a variety of intervention models (PA protocols, longitude, time of start and end of the study). Some of the clinical trials included only educational and motivational meetings or telephone calls, but no supervised exercises sessions.

In the studies that involved self-reported data (PA questionnaires), with a high number of participants (surveys, retrospective cohort studies), the rate of developed GDMs in pregnant women cohorts was 9–18.4% [161,166,167], but even reached 32% in some studies [168]. The development of GDM was correlated to PA in obese women (high BMI), with higher maternal age, a family history of diabetes and alcohol consumption [166]. Overall, the level of PA among pregnant women was reported as low [168] [and 173]. Women with GDM less frequently participated in AE and sacrificed less time for AE, but rather chose to take part in muscle strength activities (MSA). Among pregnant women meeting AE criteria, 16.5% developed GDM [168]. The Behavioural Risk Factor Surveillance System (BRFSS) study through the years of the survey (2011–2017) gathered data showing a 5-fold increase in the prevalence of GDM; in the same study, an increase in MSA but not AE was noted [161]. Low levels of PA self-reported by pregnant Asian women were shown to be associated with GDM risk, especially in obese women. A higher reported PA (more active women) was associated with 2h plasma glucose, but not in women with “sufficient” PA [167]. Total sitting time in this study was inversely correlated with plasma glucose [167].

During GDM pregnancies, the most important problems are proper weight gain control and glycaemic control in the mother and weight gain in the foetus. Higher than recommended gestational weight gain often leads to macrosomia of the foetus, which has further implications in the child’s life. Studies with PA during pregnancy show the beneficial effects of exercise on weight and glycaemic control. Pregnant women with a high risk for the development of GDM or with already developed GDM encouraged to PA reported participation in moderate exercise of various degrees (62.5–95% of participants), about 28% reported attention to vigorous activities [169,170]. The results from these studies showed better gestational weight gain control and improvements in the OGTT test [166,169,170,171]. Similar effects were achieved in clinical trials where pregnant women participated in controlled training sessions of AE or combined with MSA or stretching exercises [172,173,174,175]. Additionally, authors observed lower rates of developed GDMs [in 180–182]; there were no differences in pre-term delivery rates [174,175].

Similar to T2DM in GDM, low grade inflammation is observed. High BMI is a risk factor for GDM development but also for low grade inflammation, as increased inflammatory markers found in GDM in the blood, adipose tissue and placental tissue are usually associated with high BMI during pregnancy [86]. In obese non-GDM patients, adipokine profiles were similar to those found in obese-GDM, which further confirms the role of adiposity in inflammation and GDM [86]. Another marker of inflammation that is positively associated with GDM is CRP [176,177].

Maternal inflammation measured by CRP level was decreased in obese pregnant women encouraged to walk (goal: 11,000 steps/day) or perform other types of light intensity PA in the second and third trimesters [163,178]. hsCRP was reduced during interventions and a negative correlation between CRP and light intensity PA was noted [163,164,178]. There was also a positive association between sedentary (sitting) time measured with an accelerometer and CRP [163,164]. Active obese pregnant women showed lower CRP than non-active obese pregnant women [179]. During a single bout of cycling, active participants showed lower lipolysis, free fatty acid release and lipid oxidation than non-active pregnant women, which reflects a lower inflammatory level of active participants, as elevated levels of lipolysis, FFA and lipid oxidation are connected with an inflammatory response [179]. Data collected in the Norwegian Mother and Child Cohort Study show an inverse correlation of CRP with total recreation exercise time, but also an improvement in elevated CRP levels due to PA [166]. The strongest association between CRP and PA was noted for vigorous exercise like running, jogging/orienteering and aerobic/dance activities [166].

The GESTAFIT study revealed that inflammatory markers (IL-1β, IL-6,IL-8, IL-10, INF-γ and TNF-α) in early pregnancy are increased in normal weight women in comparison to non-pregnant women of a similar age and weight [180]. The authors concluded that pregnant women are predisposed to low grade inflammation and meeting the PA recommendations for pregnant women is associated with a decreased level of inflammatory markers in early pregnancy [180]. When patients were engaged in PA during pregnancy, a decrease of TNF-α (35th week) and increase of IL-10 at delivery were noted [181]. Additionally, IL-6 and TNF-α were lower in cord blood at delivery [181]. The beneficial effects of PA during pregnancy were not restricted to pregnancy but also to the postpartum period and influenced breast feeding. Breast milk contains fewer pro-inflammatory cytokines and more anti-inflammatory IL-10 [182]. It can be assumed that the inflammatory profile of breast milk reflects the systemic inflammatory state of the mother and is transferred to the newborn.

The presence of inflammation is obviously noted in obese GDM mothers. In the available literature, there are no studies that show focus on PA and inflammatory markers during gestation in GDM women. Although it can be expected that smaller weight gain during pregnancy, improved control of glycaemia and improved muscle fitness may decrease inflammation in GDM patients in a similar way to that seen in T2DM patients. The evidence of beneficial effects of PA on inflammatory status in healthy pregnancies in lean and obese women may also support the thesis of positive effects on inflammation in GDM women. Pregnancy alone is characterised by low grade inflammation similar to obesity or GDM and, if meeting the PA recommendations, allows the concentration of inflammatory markers to decrease in healthy pregnancy or obesity, similar effects may be expected in GDM.

## 6. Athletes with T2DM

In the past, T2DM among athletes was a rare phenomenon. Firstly, it was due to the preventive effect of PA and the relatively late-life emergence of clinically appreciable T2DM. In addition, competitive athletes were most often a group of people up to the third decade of life. The prevalence of T2DM in individuals aged 19 years and younger is 0.46 (95% CI, 0.43–0.49) per 1000 [183]. Today, in contemporary clinical practice, it is increasingly common to see “masters athletes” (MAs)—men and women over the age of 35 who participate in high-intensity and/or high-volume exercise with competitive ambitions [184]. Climstein et al. [185] investigated the prevalence of hyperglycaemia via fasting plasma glucose (FPG) in Masters athletes competing at the World Masters Games (WMG). From 8,072 MAs who completed the survey, there were 486 (males 277, females 209; mean age 55.1 ± 10.2 years) in whom FPG was measured. The mean FPG for MAs was 5.03 mmol (±1.2, 95% CI [4.9–5.1] mmol), with the majority (75.5%) of MAs reporting a normal (<5.5 mmol) FPG, followed by pre-diabetes (20.2%, >5.51 to <5.99 mmol) and abnormal (4.3%, >7.0 mmol). These results may confirm that PA protects against hyperglycaemia and that athletes are at low metabolic risk of developing T2DM [185]. This is also confirmed by the results of Laine et al. [186], who assessed the prevalence of impaired glucose regulation in male, Finnish former elite athletes. A former career as an elite athlete protected from both T2DM and IGT (impaired glucose tolerance) in later life. Furthermore, the volume of current LTPA (leisure-time physical activity) was inversely associated with the prevalence of T2DM [186].

Athletes with T2DM face many challenges to performing optimally during physical exercise. The requirement of exogenous insulin therapy greatly limits the number of active Master athletes. They are at a great risk of developing intra- and post-exercise hypoglycaemia. In this case, it is of great importance to continuously monitor blood glucose levels before and after exercise in this group [184]. In addition, it is recommended to routinely stop taking pharmaceuticals (metformin and sulfonylureas) 48 h before moderate to vigorous exercise lasting for more than 2 h to prevent incidents of lactic acidosis and hypoglycaemia [184]. Furthermore, although the mechanism has not yet been determined, several forms of CVDs, like non-ischemic myocardial fibrosis, proximal coronary artery calcification and atrial fibrillation, are more common among Masters athletes with T2DM [187,188,189]. Athletes with T2DM are at an increased risk of microvascular disease, which reduces exercise capacity, as measured by maximal oxygen consumption (VO2 max) and has important implications in the development, rehabilitation and management of musculoskeletal injuries and diseases [190]. Since physical training in T2DM results in the increased translocation of muscle GLUT4 transporters and increased capacity for insulin-stimulated glucose uptake, as well as reduced insulin resistance, physical activity is an important intervention against T2DM [191]. An important study by Knowler et al. [192] compared a 2.8-year lifestyle PA with metformin treatment and proved the efficacy of PA as it reduced the incidence of T2DM by 58% and by 31% compared to placebo, respectively.

## 7. Feasibility and Usability of PA Treatments

Although PA is a crucial element for T2DM and GDM therapies there are some complications in its implementation, especially when the training is unsupervised. The unsupervised PA sessions may be ineffective, because of poor adherence and compliance to the training regimen, mainly due to lack of knowledge, personalization and support from exercise specialists [193]. However, the unsupervised PA is generally low cost and highly accessible what seems to be of great importance in certain circumstances i.e., COVID-19 pandemic. In general, the specialists recommend avoidance of unsupervised exercise sessions due to risk of incorrect performance and increased risk of adverse effects (undesirable or harmful outcomes) [194].

Some of these issues may be resolved by usage of software apps or digital devices (activity trackers/smartwatches), which offer guidance, training plans, exercise support from health professionals i.e., in form of personalized text messages [193]. Single-arm feasibility or interventional studies show increase in feasibility and adherence to PA programmes, increase of habitual PA in individuals with access to apps or activity trackers supported with on-line sessions in T2DM patients, cancer survivals and older adults [193,194,195,196,197,198]. In study by Schoeppe et al., children, mothers and fathers using personal watches for PE support increased amount of time spent on PA and met national criteria and guidelines for PA [198]. The meta-analysis of studies concerning adverse effects showed that there is no increase in serious adverse effects due to PA, but increase of non-serious adverse effects like pain, fatigue, bursitis, oedema [194], indicating exercise training as a relatively safe intervention.

## 8. Conclusions

Physical exercise in every form is beneficial for human health and wellbeing. Appropriate amounts of PA fitted to individuals’ capabilities, both social and personal, have crucial relevance for personal health but also in terms of decreasing the social and healthcare burden due to the treatment of diabetes and its complications. Therefore, it is necessary to apply broad educational programs to increase the social awareness of the benefits of PA, as well as additional care during the treatment of patients who are prone to metabolic diseases and pregnant women to prevent the development of diabetes mellitus and GDM.

## Figures and Tables

**Table 1:** Clinical trials and parallel studies with PA in groups of T2DM subjects.

Number of Participants	Age and Gender	Type of PA	Duration of PA	Results (Inflammatory Status)	Reference
83	M, F	Walking (self-reported)	12 mo	↓CRP	[151]
100	M, F, 56–70 y.a.	AT, RT, AT + RT	4/week, 6 mo, 60 min/session	↓hs-CRP (AT, AT + RT)	[152]
48	M, F, 53.9 ± 9.9 y.a.	AT, RT, AT + RT	3/week, 3 mo, 60 min/session	↓hs-CRP, ↑TNF = α	[136]
50	M, F, 40–55 y.a.	AT: mild (55–65% HRmax) Moderate (65–75% HRmax)	3/week, 3 mo, 40 min/session	↓TNF = α, IL-2, IL-4, IL-6	[153]
24 (ob/L)ob + T2DM	M, mean 47 y.a.	AT (about 60% VO2max)	5/week, 3 mo, 60 min/session	↓IL-6, ≈CRP	[145]
10T2DM 12 c	F, 60–73 y.a.	AT (Dance classes)	2/week, 4 mo, 60 min/session	↓CRP, ↓TNF-α, (T2DM Control) ≈ IL-1β, ↑IL-1ra (C) ≈ IL-1ra (T2DM)	[154]
23 ob 12 c	F, 60–70 y.a.	RT (moderate-high intensity)	3/week, 3 mo, (10 ex. 8–10 rep. max.)	↓CRP, ↓TNF-α, ↑WBC (shortly after training)	[120]
42 T2DM 20 c	M, F, mean 52,6 y.a.	AT RT	3/week,3 mo, 40–60min/session; 3/week, 3 mo, resistance band	↓TLR4, IL-18, ↑IL-33 (but lower than c)	[131]
31 ex 33 c	M, F, mean 53.5 y.a.	AT	3/week, at least 12 sessions/2 mo	↓MDA,≈ TNF-α	[147]
33 T2DM 9 c	M 47 ± 5 y.a.	HIIT + diet	20 min/session, 3/week, 12 weeks	↓ IL-6, TNF-α	[155]
48 T2DM	M 40–65 y.a.	RT + vit.D suppl.	3/week, 12 weeks, 50 min/session	↓ IL-6, TNF-α, ≈ CRP	[146]
52 T2DM	M 39 ± 5 y.a.	AT+RT + saffron suppl.	3/week, 12 weeks, 1–1.5 h/session	↓IL-6, TNF-α, hsCRP, IL-1β, ↑IL-10	[156]
T2DM	M 40–74 y.a.	MICT vs. HIIT	12-week, twice weekly	↓FGF21, IL-6, IL-8, IL-10 and IL-18.	[138]
1239 T2DM	M, F	AT	meta-analysis	↓CRP, resistin, TNF-α, IL-6	[144]

↑—increase, ↓—decrease, M—male, F—female, AT—aerobic training, RT—resistance training, mo—months, y.a.—years of age, ob—obese, L—lean, c—control group non-diabetic, ex—exercise, rep—repetitions, WBC—white blood cells, HIIT—high intensity interval training. Studies: 145, 147, 151, 154, 155 were parallel studies; 120, 131, 136, 138, 144, 147, 152, 153, 156 were randomized controlled trials (RCTs).
